# Emergence of a Novel Plasmid-Mediated Tigecycline Resistance Gene Cluster, *tmexCD4-toprJ4*, in Klebsiella quasipneumoniae and Enterobacter *roggenkampii*

**DOI:** 10.1128/spectrum.01094-22

**Published:** 2022-07-06

**Authors:** Xun Gao, Chengzhen Wang, Luchao Lv, Xiaotong He, Zhongpeng Cai, Wanyun He, Tong Li, Jian-Hua Liu

**Affiliations:** a College of Veterinary Medicine, Key Laboratory of Zoonosis of Ministry of Agricultural and Rural Affairs, Guangdong Provincial Key Laboratory of Veterinary Pharmaceutics Development and Safety Evaluation, South China Agricultural University, National Risk Assessment Laboratory for Antimicrobial Resistant of Microorganisms in Animals, Guangzhou, China; b Guangdong Laboratory for Lingnan Modern Agriculture, Guangzhou, China; China Agricultural University

**Keywords:** RND efflux pump gene cluster, tigecycline, plasmid, *Enterobacteriaceae*

## Abstract

The occurrence of transferable tigecycline resistance determinants, *tmexCD1-toprJ1*, *tmexCD2-toprJ2*, *tmexCD3-toprJ1b*, and multiple *tet*(A) and *tet*(X) variants, presents an unprecedented challenge to clinical therapeutic options. *tmexCD-toprJ*-like gene clusters can mediate multidrug resistance and have been detected in a variety of bacteria. Here, we characterized the fourth *tmexCD-toprJ*-like gene cluster, *tmexCD4-toprJ4*, identified on untypeable plasmids of Klebsiella quasipneumoniae and Enterobacter roggenkampii isolated from chicken meat and environmental samples from farm markets, respectively. TMexCD4-TOprJ4 was closely related (92 to 99% amino acid identity) to TMexCD1-TOprJ1, TMexCD2-TOprJ2, and TMexCD3-TOprJ1. Phylogenetic analysis revealed that *tmexCD4-toprJ4* was not in the same branch as the other three variants. Expression of *tmexCD4-toprJ4* increased tigecycline efflux in Escherichia coli and resulted in a 4- to 8-fold increase in MICs of tigecycline in E. coli and Klebsiella pneumoniae. Moreover, *tmexCD4-toprJ4* can act synergistically with its upstream gene *tet*(A) to reduce the susceptibility of E. coli and K. pneumoniae strains to tigecycline. The *tmexCD4-toprJ4-*containing plasmid is a novel plasmid type and can be transferred to E. coli and K. pneumoniae only via electrotransformation. The increasing emergence of plasmid-mediated tigecycline resistance gene clusters suggests that the spread of *tmexCD-toprJ*-like gene clusters requires widespread attention.

**IMPORTANCE** The plasmid-mediated tigecycline resistance gene cluster *tmexCD1-toprJ1* and other variants have been detected in a variety of strains from multiple sources, including human-derived strains. In addition to tigecycline, these *tmexCD-toprJ*-like gene clusters reduce susceptibility of the host strain to many other antimicrobials. Here, we identified *tmexCD4-toprJ4* in *K. quasipneumoniae* and *E. roggenkampii*, suggesting that this gene cluster is already present in the human-associated environment and the risk of transmission to humans is increased. Monitoring tigecycline-resistant Gram-negative bacteria is essential for understanding and addressing the spread of this gene cluster in agriculture and health care.

## OBSERVATION

Tigecycline is a last-resort antibiotic used to treat severe clinical infections caused by multidrug-resistant (MDR) bacteria (1). However, the widespread use of tigecycline has led to the evolution of resistance, which reduces therapeutic effectiveness. Notably, the recent emergence of plasmid-mediated *tet*(X) variants (2) and transferable resistance-nodulation-division (RND) family efflux pump gene clusters (3) that confer resistance to tigecycline has increased the challenges of controlling tigecycline resistance. Since the emergence of the RND family efflux pump resistance gene cluster *tmexCD1-toprJ1* in Klebsiella pneumoniae, two additional *tmexCD-toprJ*-like gene clusters, *tmexCD2-toprJ2* (4) and *tmexCD3-toprJ1b* (5), have also been identified.

*tmexCD1-toprJ1*, which confers multidrug resistance, has been detected in K. pneumoniae (1), Klebsiella quasipneumoniae (6), Raoultella planticola (1), Klebsiella oxytoca (1), and Enterobacter cloacae and might be transferred by site-specific integrases (1) or IS*26* (6). *tmexCD1-toprJ1* has been detected in humans, animals, food, and sewage. *tmexCD2-toprJ2* has been found in K. pneumoniae, Citrobacter freundii, Raoultella ornithinolytica, Klebsiella variicola, *K. quasipneumoniae*, and Klebsiella michiganensis (7) isolated from environmental or clinical samples (4). *tmexCD2-toprJ2* may be mobilized by the XerD-like recombinase system (4). Subsequently, *tmexCD3-toprJ1b* was found in Pseudomonas aeruginosa and Proteus mirabilis isolated from chicken fecal samples and is located in an integrative and conjugative element (ICE) (5). The three gene clusters could mediate the 4- to 16-fold decrease in tigecycline susceptibility of the host bacteria. In this study, we report a novel plasmid-mediated *tmexCD-toprJ*-like gene cluster, *tmexCD4-toprJ4*, in *K. quasipneumoniae* obtained from chicken meat and Enterobacter roggenkampii obtained from a farm market environment in China.

From May 2019 to July 2021, 109 chicken meat samples and 128 environmental samples were collected from farm markets in Guangzhou, China. All samples were selected on MacConkey agar plates supplemented with 4 mg/L tigecycline. Colonies with different morphologies were selected and screened for *tmexCD1-toprJ1* by PCR using specific primers (see Table S1 in the supplemental material). Nine (8.2%) *tmexCD1-toprJ1*-positive strains and two *tmexCD1-toprJ1*-like strains (GLW9C22 and GD21SC1505) were identified. GLW9C22 was isolated from a chicken meat sample and showed resistance to florfenicol, apramycin, and several tetracyclines including tigecycline (32 μg/mL) (Table S2). GD21SC1505, which was recovered from an environmental sample, showed resistance to florfenicol, colistin, and tigecycline (16 μg/mL). Meanwhile, the MIC of tigecycline decreased to 0.5 μg/mL for both strains in the presence of the efflux pump inhibitor 1-(1-naphthylmethyl)-piperazine (NMP) (Table 1).

**TABLE 1:** Overall features of strains in this study

Strain	Bacterial species	Plasmid or chromosome	Plasmid type	Size (bp)	Resistance gene(s)	MIC of tigecycline (+NMP) (mg/L)
GLW9C22	*K. quasipneumoniae*	Chromosome		5,129,993	*oqxAB*, *fosA*	32 (0.25)
		pHNLW22-1	IncFIB(K)	259,761	*aac(3)-IV*, *aph(4)-Ia aph(3′)-Ia*, *floR*, *aadA2*, *aph(3′)-Ia*, *tet*(A)	
		pHNLW22-2		34,729	*tmexCD4-toprJ4*, *tet*(A), *floR*	
		pHNLW22-3		4,150		
GD21SC1505	*E. roggenkampii*	Chromosome		4,780,540	*bla*_MIR-6_	16 (0.25)
		pHN21SC1505-1	IncFII	90,290		
		pHN21SC1505-2	IncX2	40,695	*qnrS1*, *tet*(A)	
		pHN21SC1505-3		34,729	*tmexCD4-toprJ4*, *tet*(A), *floR*	
		pHN21SC1505-4	IncP6	27,664		
		pHN21SC1505-5		5,027		
BW25113	E. coli					0.125 (0.125)
BW25113-pHNLW22-2	E. coli	pHNLW22-2		34,729	*tmexCD4-toprJ4*, *tet*(A), *floR*	4 (0.25)
BW25113-pHSG575	E. coli	pHSG575				0.125 (0.125)
BW25113-pHSG575-tet(A)	E. coli	pHSG575-tet(A)			*tet*(A)	0.25 (0.25)
BW25113-pHSG575-tmexCD4-toprJ4	E. coli	pHSG575-tmexCD4-toprJ4			*tmexCD4-toprJ4*	1 (0.25)
BW25113-pHSG575-tet(A)-tmexCD4-toprJ4	E. coli	pHSG575-tet(A)-tmexCD4-toprJ4			*tmexCD4-toprJ4*, *tet*(A)	2 (0.25)
BW25113Δ*acrAB*	E. coli					0.03 (0.03)
BW25113Δ*acrAB*-pHNLW22-2	E. coli	pHNLW22-2		34,729	*tmexCD4-toprJ4*, *tet*(A), *floR*	4 (0.25)
BW25113Δ*acrAB*-pHSG575	E. coli	pHSG575				0.03 (0.03)
BW25113Δ*acrAB*-pHSG575-tet(A)	E. coli	pHSG575-tet(A)			*tet*(A)	0.06 (0.06)
BW25113Δ*acrAB*-pHSG575-tmexCD4-toprJ4	E. coli	pHSG575-tmexCD4-toprJ4			*tmexCD4-toprJ4*	1 (0.125)
BW25113Δ*acrAB*-pHSG575-tet(A)-tmexCD4-toprJ4	E. coli	pHSG575-tet(A)-tmexCD4-toprJ4			*tmexCD4-toprJ4*, *tet*(A)	2 (0.125)
AH58I	K. pneumoniae					0.5 (0.5)
AH58I-pHNLW22-2	K. pneumoniae	pHNLW22-2		34,729	*tmexCD4-toprJ4*, *tet*(A), *floR*	16 (0.5)
AH58I-pHSG575	K. pneumoniae	pHSG575				0.5 (0.5)
AH58I-pHSG575-tet(A)	K. pneumoniae	pHSG575-tet(A)			*tet*(A)	1 (1)
AH58I-pHSG575-tmexCD4-toprJ4	K. pneumoniae	pHSG575-tmexCD4-toprJ4			*tmexCD4-toprJ4*	4 (0.5)
AH58I-pHSG575-tet(A)-tmexCD4-toprJ4	K. pneumoniae	pHSG575-tet(A)-tmexCD4-toprJ4			*tmexCD4-toprJ4*, *tet*(A)	8 (0.5)

The complete genomic DNA data of GLW9C22 and GD21SC1505 were generated by the combination of Illumina HiSeq platforms and Nanopore MinION, followed by assembly using Unicycler version 0.4.3.8. The results revealed that GLW9C22 belonged to *K. quasipneumoniae* and harbored a 5,129,993-bp chromosome and three plasmids. Moreover, GLW9C22 carried several known acquired antibiotic resistance genes, including *aac(3)-IV*, *aph(4)-Ia*, *aph(3′)-Ia*, *floR*, *aadA2*, *aph(3′)-Ia*, and *tet*(A). GD21SC1505 belonged to *E. roggenkampii* and harbored a 4,780,540-bp chromosome and five plasmids (Table 1). GD21SC1505 harbored the resistance genes *bla*_MIR-6_, *qnrS1*, *tet*(A), and *floR*. Although GD21SC1505 showed resistance to colistin, this strain did not carry colistin resistance-related genes, and no mutations in related genes were detected on the chromosome; therefore, the reason for resistance needs to be further investigated. *K. quasipneumoniae* branched from K. pneumoniae as a new bacterial species in 2014 (8). This species can persist in hospitalized patients and in the hospital environment for a long time and can spread between patients and sink drains. Moreover, *K. quasipneumoniae* has acquired multiple clinically important resistance genes, including carbapenem and colistin resistance genes (9). *E. roggenkampii* is a type of E. cloacae complex species (10) that frequently carries *mcr-10* and has been detected in humans (11), chickens (12), and dogs (13).

**FIG 1:**
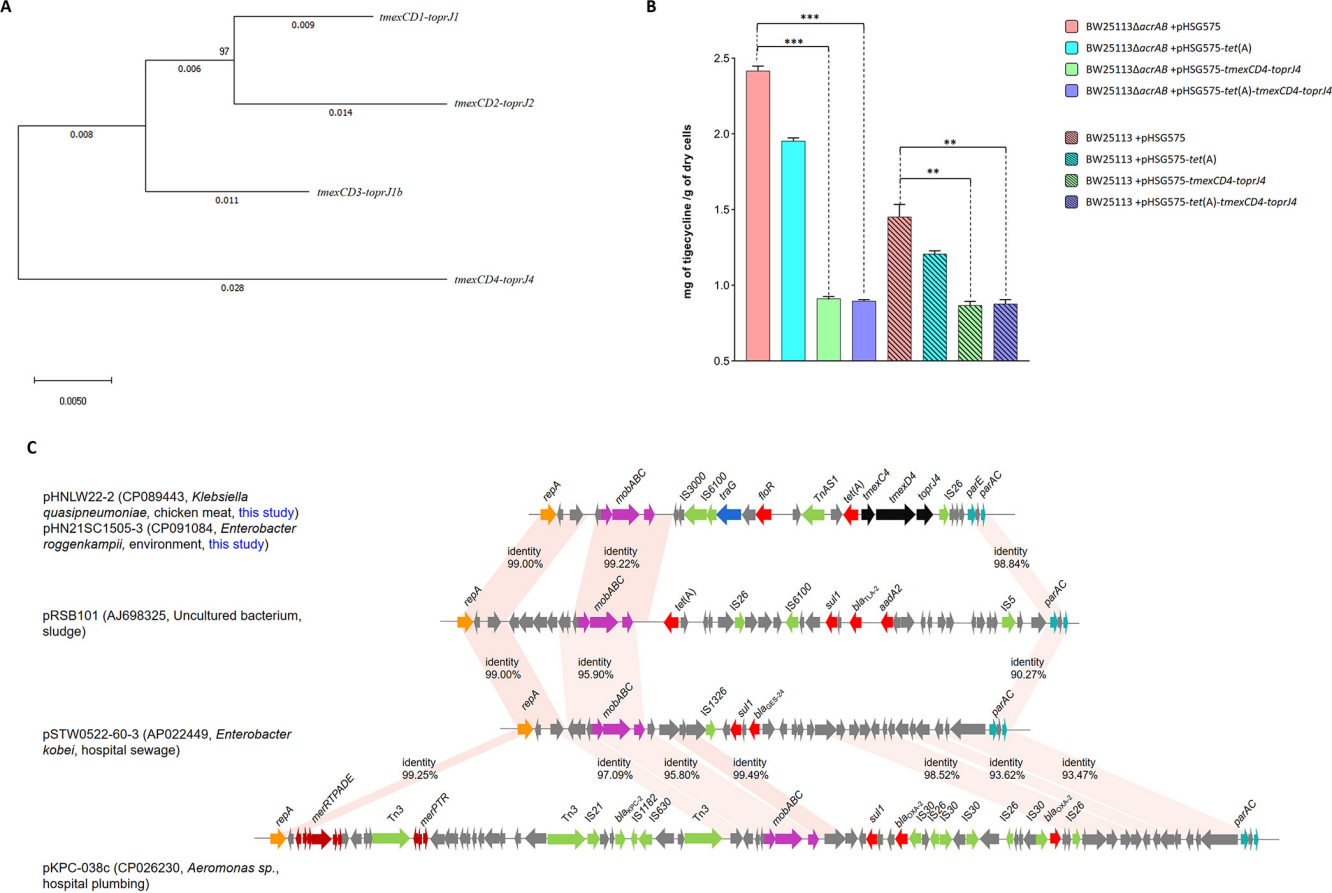
(A) The phylogenetic tree of sequences of *tmexCD4-toprJ4* and three *tmexCD-toprJ* alleles. The nucleotide sequences of three *tmexCD-toprJ* alleles were obtained from NCBI and aligned with *tmexCD4-toprJ4* using ClusterX. Then, the evolutionary tree was generated using the neighbor-joining method with MegaX. (B) Tigecycline intracellular accumulation levels. Tigecycline accumulation in E. coli BW25113 and BW25113Δ*acrAB* carrying pHSG575, pHSG575-tet(A), pHSG575-tmexCD4-toprJ4, or pHSG575-tet(A)-tmexCD4-toprJ4 was determined at the 20-min incubation point. Each column shows the mean and the standard deviation for three replicates. Student’s *t* tests were performed to statistically analyze the data. **, *P* < 0.01; ***, *P* < 0.005. (C) Comparison of plasmid carrying *tmexCD4-toprJ4* with related plasmids. The lengths and directions of genes are shown by arrows labeled by gene names. Genes with different functions are represented by different colors. Horizontal lines represent the plasmid backbone.

The *tmexCD1-toprJ1-*like gene cluster was identified in plasmids pHNLW22-2 and pHN21SC1505-3. Compared with *tnfxB1*-*tmexCD1-toprJ1*, this gene cluster lacks the upstream regulator gene *tnfxB*, thereby forming the structure of *tmexC-tmexD-toprJ*. At the nucleotide level, it displays high homology with *tmexCD1-toprJ1*, *tmexCD2-toprJ2*, and *tmexCD3-toprJ1b* (Table S3). Moreover, at the amino acid level, the proteins encoded by this gene cluster shared 98.97%, 96.84%, and 92.26% identity with TMexC1, TMexD1, and TOprJ1; 97.68%, 98.56%, and 92.47% identity with TMexC2, TMexD2, and TOprJ2; and 98.20%, 98.09%, and 92.26% identity with TMexC3, TMexD3, and TOprJ1, respectively. Phylogenetic analysis of this gene cluster with related nucleotide sequences revealed that it belongs in a separate branch (Fig. 1A). Therefore, we assigned this gene cluster to a new allele, *tmexCD4-toprJ4.*

To verify the role of *tmexCD4-toprJ4* in antimicrobial resistance, a recombinant plasmid, pHSG575-tmexCD4-toprJ4, was constructed. Compared to the empty vector pHSG575, it showed an 8-fold increase in the MICs of tigecycline in both host strains, Escherichia coli BW25113 and K. pneumoniae AH58I. Reduced susceptibility was also observed for ciprofloxacin (4-fold increase), cefquinome (8-fold increase), and apramycin (2-fold increase). Therefore, like *tmexCD1-toprJ1*, *tmexCD4-toprJ4* is also a multidrug resistance gene cluster. However, the MIC of tigecycline conferred by this recombinant plasmid was 8-fold lower in recipient K. pneumoniae AH58I than in the wild-type strain GLW9C22 (Table 1). Further sequence analysis revealed that a major facilitator superfamily transporter gene, *tet*(A), was present upstream of *tmexCD4-toprJ4*. Hence, to investigate the effect of Tet(A) on the function of TMexCD4-TOprJ4, two recombinant plasmids, pHSG575-tet(A)-tmexCD4-toprJ4 and pHSG575-tet(A), were constructed by cloning and ligating the *tet*(A) sequence as well as the intergenic sequence between *tmexCD4-toprJ4* and *tet*(A). Relative to the strain with the empty vector, pHSG575-tet(A) and pHSG575-tet(A)-tmexCD4-toprJ4 increased tigecycline MIC by 2-fold and 16-fold in BW25113 and AH58I, respectively. To further determine the antimicrobial-resistant phenotype mediated by TMexCD4-TOprJ4, an extremely sensitive host strain, BW25113Δ*acrAB* (an *acrAB* knockout mutant [14]), was used to express *tmexCD4-toprJ4* and *tet*(A). The results also revealed that TMexCD4-TOprJ4 mediated tigecycline resistance with a 32-fold increase in tigecycline MIC. Expression of both *tmexCD4-toprJ4* and *tet*(A) resulted in a 64-fold increase in tigecycline MIC, suggesting a synergistic effect of TMexCD4-TOprJ4 and TetA. Therefore, the coexistence of *tmexCD4-toprJ4* and *tet*(A) allows the host strain to survive better under drug selection pressure. To further check the expression of *tmexCD4-toprJ4* and *tet*(A), the transcriptional levels of *tmexC4*, *tmexD4*, *toprJ4*, and *tet*(A) were measured. Among strains carrying *tet*(A), *tmexCD4-toprJ4*, or *tet*(A)-*tmexCD4-toprJ4*, the expression of *tmexCD4-toprJ4* and that of *tet*(A) were at a similar level. However, expression levels of these genes in BW25113Δ*acrAB* were higher than those in BW25113 (Fig. S1), which might be due to the changes of the *acrAB*-related regulators caused by the *acrAB* deletion (15). But the specific reasons need to be further analyzed.

To confirm that TMexCD4-TOprJ4 acts as an efflux pump and acts synergistically with Tet(A), we determined the intracellular accumulation of tigecycline in E. coli carrying *tet*(A), *tmexCD4-toprJ4*, *tet*(A)-*tmexCD4-toprJ4*, or the control plasmid pHSG575. BW25113 and BW25113Δ*acrAB* were employed as sensitive hosts to assess efflux efficiency. Compared with bacterial cells carrying the empty plasmid pHSG575, both host strains expressing *tmexCD4-toprJ4* significantly decreased the intracellular amount of tigecycline, whereas cells carrying *tet*(A) also showed slightly lower intracellular drug concentrations (Fig. 1B). It is worth noting that the intracellular drug concentration in BW25113Δ*acrAB*-*tmexCD4-toprJ4* was similar to that in BW25113-*tmexCD4-toprJ4*, implying that TMexCD4-TOprJ4 independently functioned as a drug transporter. Furthermore, we observed an almost equal concentration of intracellular tigecycline accumulation in cells carrying *tmexCD4-toprJ4* or *tet*(A)-*tmexCD4-toprJ4* in both bacterial hosts, demonstrating the prominent role of TMexCD4-TOprJ4 in the efflux of tigecycline. According to these results, it could be found that the resistance phenotype and drug efflux capacity conferred by *tmexCD4-toprJ4* in BW25113 or BW25113Δ*acrAB* are at similar levels, indicating that the function of *tmexCD4-toprJ4* is independent of *acrAB*. However, there are differences in the drug efflux capacity between BW25113-pHSG575-tet(A) and BW25113Δ*acrAB*-pHSG575-tet(A), indicating that as previously reported, the tigecycline efflux function of *tet*(A) is *acrAB* dependent (16, 17).

Further whole-genome sequencing analysis showed that pHNLW22-2 and pHN21SC1505-3 were both 34,729-bp in length and had a GC content of 60.04%, differing by only five single nucleotide polymorphisms (SNPs). Therefore, pHNLW22-2 was used as the test plasmid in subsequent experiments. A 15-day antibiotic-free stability test indicated that pHNLW22-2 was stable in the original strain as well as in E. coli BW25113 (Fig. S2), suggesting that this small plasmid can replicate and remain stable in subcultures. pHNLW22-2 failed to be transferred from donor strain GLW9C22 into recipient E. coli J53 via the conjugation assay at 30°C, 37°C, and 42°C because of the lack of a functional conjugative system (Fig. 1C). However, it was transformed into BW25113, BW25113Δ*acrAB*, and AH58I by electrotransformation, and the tigecycline MIC was increased by 32-fold, 128-fold, and 32-fold, respectively (Table 1). This plasmid was untypeable using PlasmidFinder, and the complete structure of pHNLW22-2 included a replication system (*repA*), a partitioning system (*parA*, *parC*), a mobilization system (*mobABC*), and an ~21-kb variable region (Fig. 1C). BLASTn results showed that the backbone of pHNLW22-2 was highly similar to the untypeable plasmid pRSB101, with 90% coverage and 99.05% identity, which were identified from uncultured bacteria in sludge. It also exhibited high levels of homology to the backbone of *bla*_GES-24_-carrying plasmid pSTW0522-60-3 (Enterobacter kobei, Japan, sewage, AP022449) and *bla*_KPC-2_-carrying plasmid pKPC-038c (*Aeromonas* sp., United States, wastewater, CP026230), with 81% coverage and 98.8% identity (Fig. 1C). The main differences among these plasmids are the different resistance genes or heavy metal resistance genes and the diversity of insertion sequences carried in the variable region. Previous studies indicated that the replication region and partitioning system of pRSB101 (pHNLW22-2-like plasmid) were highly similar to those of plasmids isolated from environmental bacteria and phytopathogenic bacteria (such as *Xanthomonas* and *Aeromonas*), suggesting that pHNLW22-2-like plasmids likely originated from environmental bacteria (18). In addition, the pRSB101 plasmid can be mobilized to the recipient strain by a self-transmissible helper plasmid (IncP-1α plasmid RP4), and pHNLW22-2 and pHNLW22-2-like plasmids have spread to various bacterial species and may belong to broad-host-range plasmids (18). Therefore, the broad host range and transferable characteristics of pHNLW22-2 will accelerate the transmission of *tet*(A)*-tmexCD4-toprJ4* gene clusters in different bacterial species. In addition to *tmexCD4-toprJ4*, more antimicrobial resistance genes originating from environmental bacteria might be captured by the pHNLW22-2-like plasmid and then transferred to other bacteria, further exacerbating the increasingly serious challenges of antimicrobial resistance.

Further genetic context analysis of *tmexCD4-toprJ4* revealed a Tn*As1-hp-tet*(A)-*tmexC4-tmexD4-toprJ4*-IS*26* structure (Fig. 1C), which was different from the genetic structures of three previously reported *tmexCD-toprJ-*like gene clusters associated with site-specific integrases, IS*26* (6) or ICE (5). Although two mobile elements, Tn*As1* and IS*26*, were present on the flanks of *tmexCD4-toprJ4*, there was no evidence that Tn*As1* and IS*26* could mediate the transfer of *tmexCD4-toprJ4*. Therefore, we hypothesized that *tmexCD4-toprJ4* may have been transferred into this plasmid through multiple insertional recombination events.

In conclusion, we are the first to report a novel plasmid-mediated RND family efflux pump, *tmexCD4-toprJ4*, carried by an untypeable plasmid in *K. quasipneumoniae* and *E. roggenkampii* isolated from chicken meat and environmental samples from farm markets*. tmexCD4-toprJ4* significantly differs from the previously reported tigecycline resistance efflux pump clusters at both the nucleotide and amino acid levels, as well as in the genetic structure. TMexCD4-ToprJ4 mediated low-level tigecycline resistance and exhibited a synergistic effect with Tet(A) in reducing tigecycline susceptibility. Therefore, screening for the *tmexCD-toprJ-*like gene cluster should be urgently included in the surveillance of tigecycline-resistant Gram-negative pathogens in animals, humans, and the environment.

### Data availability.

The complete sequences of the GLW9C22 chromosome and four plasmids and the GD21SC1505 chromosome and five plasmids were submitted to GenBank with accession numbers CP089441 to CP089444 and CP091081 to CP091086, respectively.
