# Supplementary material for: Emergence of a Novel Plasmid-Mediated Tigecycline Resistance Gene Cluster, tmexCD4-toprJ4, in Klebsiella quasipneumoniae and Enterobacter roggenkampii
Source: Microbiol Spectr. 2022 Jul 6;10(4):e01094-22. doi: 10.1128/spectrum.01094-22 (PMC9431256; doi:10.1128/spectrum.01094-22)
Supplement: Supplemental file 1 — Supplemental material. Download spectrum.01094-22-s0001.pdf, PDF file, 0.3 MB [file spectrum.01094-22-s0001.pdf]

Supplementary Materials

Table S1. Primers used in this study

Primer	Sequence(5'-3')
tmexD-F	CAGCCAGGACTACAACTTC
tmexD-R	TAGAGGAACTTCGGATTGC
16s-F	TGTAGCGGTGAAATGCGTAGA
16s-R	CACCTGAGCGTCAGTCTTCGT
qtmexC4-F	CGCTGAGCACATCGACTTCA
qtmexC4-R	CTTTTTCCGTGATCTCCTATT
qtmexD4-F	TGACGGTGATCTCGGCTATG
qtmexD4-R	CTCGGTGACCAGCGTCATC
qtoprJ4-F	TTGAGCGACGGCAGACGG
qtoprJ4-R	TGGATACCGCACTGAACAACAA
qtet(A)-F	CCAACAGACCCCTGATCGTAA
qtet(A)-R	ACGTCGTTCGAGTGAACCAGAT

Table S2. MICs (mg/L) of strains used in this study

	TIG (+NMP）	DOX	TET	IMP	CIP	AMP	CAZ	CQM	CTX	AMK	APR	GEN	NEO	STR	FFC	CL
GLW9C22	32 (0.25)	64	>128	0.125	0.03	64	0.125	0.25	0.06	1	>128	4	8	8	>128	0.25
GD21SC1505	16 (0.25)	64	>128	0.125	0.5	>128	4	2	1	1	4	2	1	4	>128	>64
BW25113	0.125 (0.125)	0.125	0.25	0.03	0.002	1	0.03	0.008	0.015	0.25	1	0.125	4	1	1	0.125
BW25113-pHNLW22-2	4 (0.25)	16	128	0.03	0.008	1	0.03	0.008	0.015	0.25	1	0.125	4	1	4	0.125
BW25113--pHSG575	0.125 (0.125)	0.125	0.25	0.03	0.002	1	0.03	0.008	0.015	0.25	1	0.125	4	1	1	0.125
BW25113-pHSG575-tet(A)	0.25 (0.25)	4	64	0.03	0.002	1	0.03	0.008	0.015	0.25	1	0.125	4	1	1	0.125
BW25113-pHSG575-tmexCD4-toprJ4	1 (0.25)	8	64	0.03	0.008	1	0.03	0.06	0.015	0.25	2	0.125	4	1	1	0.125
BW25113-pHSG575-tet(A)-tmexCD4-toprJ4	2 (0.25)	16	128	0.03	0.008	1	0.03	0.06	0.015	0.25	2	0.125	4	1	1	0.125
BW25113Δ*acrAB*	0.03 (0.03)	0.125	0.25	0.03	0.002	1	0.03	0.008	0.015	0.25	1	0.125	4	1	1	0.125
BW25113Δ*acrAB*-pHNLW22-2	4 (0.25)	16	128	0.03	0.002	1	0.03	0.008	0.015	0.25	1	0.125	4	1	4	0.125
BW25113Δ*acrAB*-pHSG575	0.03 (0.03)	0.125	0.25	0.03	0.002	1	0.03	0.008	0.015	0.25	1	0.125	4	1	1	0.125
BW25113Δ*acrAB*-pHSG575-tet(A)	0.06 (0.06)	4	64	0.03	0.002	1	0.03	0.008	0.015	0.25	1	0.125	4	1	1	0.125
BW25113Δ*acrAB*-pHSG575-tmexCD4-toprJ4	1 (0.125)	8	64	0.03	0.008	1	0.03	0.06	0.015	0.25	2	0.125	4	1	1	0.125
BW25113Δ*acrAB*-pHSG575-tet(A)-tmexCD4-toprJ4	2 (0.125)	16	128	0.03	0.008	1	0.03	0.06	0.015	0.25	2	0.125	4	1	1	0.125
AH58I	0.5 (0.5)	2	4	8	32	>128	>128	128	>128	1	>128	4	1	16	>128	0.25
AH58I-pHNLW22-2	16 (0.5)	64	>128	8	32	>128	>128	128	>128	1	>128	4	1	16	>128	0.25
AH58I-pHSG575	0.5 (0.5)	2	4	8	32	>128	>128	128	>128	1	>128	4	1	16	>128	0.25
AH58I-pHSG575-tet(A)	1 (1)	16	>128	8	32	>128	>128	128	>128	1	>128	4	1	16	>128	0.25
AH58I-pHSG575-tmexCD4-toprJ4	4 (0.5)	32	>128	8	32	>128	>128	128	>128	1	>128	4	1	16	>128	0.25
AH58I-pHSG575-tet(A)-tmexCD4-toprJ4	8 (0.5)	32	>128	8	32	>128	>128	128	>128	1	>128	4	1	16	>128	0.25

TIG, tigecycline; TET, tetracycline; DOX, doxycycline; AMP, ampicillin; CQM, cefquinome; STR, streptomycin; NEO, neomycin; FFC, florfenicol; CL, colistin; IPM, imipenem; CIP, ciprofloxacin; AMI, amikacin; GEN, gentamycin; APR, ampramycin; CTX, cefotaxime; CAZ, ceftazidime.

Table S3. Nucleotide and amino acid identity between *tmexCD4-toprJ4* and its homology genes

Gene (protein)	*tmexC1* (TMexC1)	*tmexD1* (TMexD1)	*toprJ1* (TOprJ1)	*tmexC2* (TMexC2)	*tmexD2* (TMexD2)	*toprJ2* (TOprJ2)	*tmexC3* (TMexC3)	*tmexD3* (TMexD3)	*toprJ1b* (TOprJ1b)
tmexC4 (TMexC4)	97.51% (98.975%)			96.05% (97.68%)			97.42% (98.20%)		
tmexD4 (TMexD4)		95.95% (96.84%)			96.62% (98.56%)			97.13% (98.09%)	
toprJ4 (TOprJ4)			90.32% (92.26%)			90.39% (92.47%)			90.32% (92.26%)

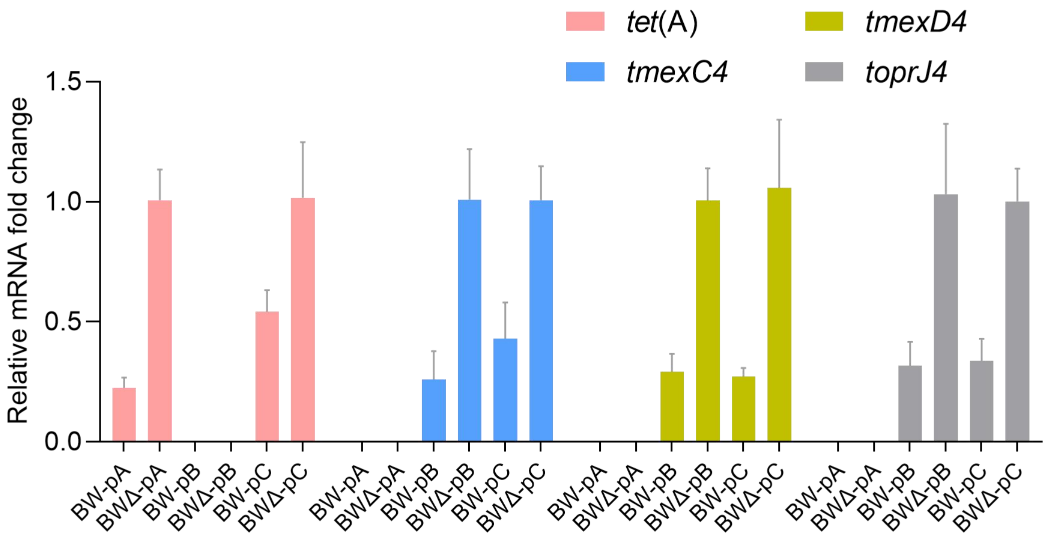

Fig. S1 The relative mRNA fold changes of *tet*(A) and *tmexCD4-toprJ4* genes. pA represents pHSG575-tet(A), pB represents pHSG575-tmexCD4-toprJ4, pC represents pHSG575-tet(A)-tmexCD4-toprJ4, BW represents BW25113, BWΔ represents BW25113Δ*acrAB*.

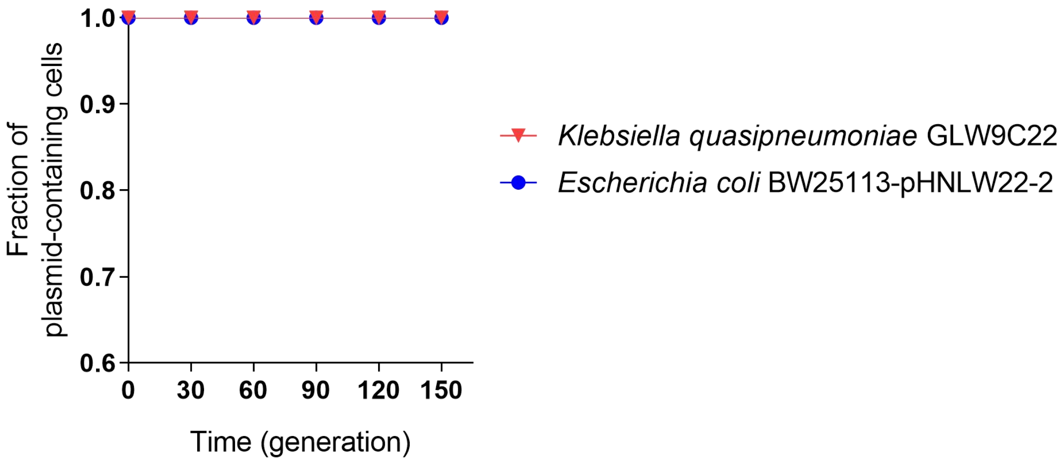

Fig. S2 Stability of pHNLW22-2 in *Klebsiella quasipneumoniae* GLW9C22 and *E. coli* BW25113.

Materials and methods

Bacteria isolate and antimicrobial susceptibility test

During May 2019 to July 2021, a total of 109 chicken meat samples and 128 environment samples were collected from farm markets located in Guangzhou, China. All the samples were selected using MacConkey agar plates supplied with 4 mg/L tigecycline. PCR amplification was conducted to screen for *tmexCD1-toprJ1-like* gene cluster using specific primers (Table S1) and PCR products were verified by sanger sequencing. The minimum inhibitory concentrations (MICs) for *tmexCD1-toprJ1-like* positive strains were determined using the broth microdilution method. *Escherichia coli* ATCC 25922 was served as the quality control strain and MICs results were interpreted in accordance with EUCAST standards (http://www.eucast.org/clinical_breakpoints/). Tigecycline MICs were also measured in the presence of the efflux pump inhibitor 1-(1-naphthylmethyl)-piperazine (NMP) with the final concentration of 75 mg/L.

Conjugation experiments

Conjugation assays were performed using *K. quasipneumoniae* GLW9C22 as the donor strain and *E. coli* J53 as the recipient strain at 30, 37 and 42 degrees, respectively. Transconjugants were selected using MacConkey agar plates supplemented with sodium azide (150 mg/L) and tigecycline (2 mg/L). Plasmids were extracted using TIANprep Rapid Mini Plasmid kit (TIANGEN) and electrotransfered to recipients, including *E. coli* BW25113, BW25113Δ*acrAB* (a *acrAB* knock out mutant (1)), and carbapenem-resistant *K. pneumoniae* AH58I. Transformants were screened using Luria Bertani (LB) agar plates containing tigecycline (2 mg/L).

Whole genome sequencing and analysis

Genomic DNA was extracted by HiPure Bacteria DNA Kit (Magen, China), and was sequenced by using Illumina NovaSeq 6000 and minION platform (Nanopore, Oxford, UK). Hybrid assembly was performed by using Unicycler version 0.4.8. Antimicrobial resistance genes and IS elements were identified by Resfinder 4.1

(https://cge.cbs.dtu.dk/services/ResFinder/) and ISfinder (https://isfinder.biotoul.fr/), respectively.

Functional cloning and tigecycline accumulation assay

DNA fragment containing *tmexCD4-toprJ4, tet*(A)-*tmexCD4-toprJ4* or *tet*(A) were amplified using primers listed in Table S1 and were ligated into pHSG575 using the Seamless Assembly Cloning Kit (Clone Smarter Technologies Inc., Houston, TX, USA), to construct recombinant plasmids, pHSG575-tmexCD4-toprJ4, pHSG575-tet(A)-tmexCD4-toprJ4, and pHSG575-tet(A). Then the three recombinant plasmids were transformed into BW25113, BW25113Δ*acrAB*, and AH58I.

Tigecycline accumulation assay was assessed as described previously, with several modifications. Briefly, overnight culture of each strain were grown in LB broth until reached in the exponential-phase (OD600 to 0.5), then the bacterial cells was centrifuged at 4000 g for 10 min at 4 °C. Enrich bacterial cells were suspended in the phosphate-buffered saline buffer to the density at 600 nm of 3.0. Then glucose was added to the cell suspension to 100 mM and for 20 min at 37 °C to energize the efflux pump. After the addition of tigecycline to final concentration of 100 mg/L, the 1-ml cell samples were collected, washed, lysed, and detected with high performance liquid chromatography at 20 min drug incubation time point, which were the same as described before.

RNA extraction and RT-qPCR

Total RNA of BW25113-pHSG575-tet(A)-tmexCD4-toprJ4 and BW25113Δ*acrAB*-pHSG575-tet(A)-tmexCD4-toprJ4 was extracted by using the Hipure Bacterial RNA Kit (Magen, China). Reverse transcription was carried out with 1 μg RNA using TB Green Premix Ex Taq™ II (Takara, Japan). Targets of around 110 bp were amplified with primers qtmexC4-F/R, qtmexD4-F/R, qtoprJ4-F/R, and qtet(A)-F/R by qPCR, while 16S genes was used as internal control with primers 16s-F/R. Relative expression was estimated by the 2-ΔΔCt method. The experiment was performed with three biological replicates.

References

1. Datsenko KA, Wanner BL. 2000. One-step inactivation of chromosomal genes in *Escherichia coli* K-12 using PCR products. Proc Natl Acad Sci U S A 97(12):6640. https://hub.pubmedplus.com/10.1073/pnas.120163297.
